# Engineered targeted Ce-based MOF nanozymes for ROS scavenging and inflammatory Reprogramming in chronic pancreatitis

**DOI:** 10.1016/j.mtbio.2026.102811

**Published:** 2026-01-21

**Authors:** Yongkang Lai, Yongliang Ouyang, Xiaojing Yin, Tao Yu, Jianhua Wan, Xueyang Li, Yi Hu, Xu Shu, Huan Wang

**Affiliations:** a Department of Gastroenterology, Jiangxi Provincial Key Laboratory of Digestive Diseases, Jiangxi Clinical Research Center for Gastroenterology, Digestive Disease Hospital, The First Affiliated Hospital, Jiangxi Medical College, Nanchang University, Nanchang, 330006, China; b Shanghai Key Lab. of D&A for Metal Functional Materials, School of Materials Science & Engineering, Tongji University, Shanghai, 201804, China; c Department of Gastroenterology and Hepatology, Jiangxi Provincial People's Hospital, The First Affiliated Hospital of Nanchang Medical College, Nanchang, 330006, Jiangxi Province, China

**Keywords:** Chronic pancreatitis, Reactive oxygen species, Metal–organic framework, Targeted therapy, Nanozyme

## Abstract

Chronic pancreatitis (CP) is a lifelong progressive fibrotic inflammatory disorder for which no effective cure is currently available. Persistent and recurrent inflammatory stimulation induced by reactive oxygen species (ROS) is a key driver of pancreatic fibrogenesis, making oxidative stress a promising therapeutic target to halt disease progression. In this study, we developed a nanosystem, HC@CeMOF, consisting of a small-sized cerium-based metal–organic framework (CeMOF) core loaded with curcumin and coated with hyaluronic acid (HA), enabling precise targeting of inflamed pancreatic tissue. HC@CeMOF exhibits a small-sized particle size along with favorable cellular and biological safety profiles. Once administered *in vivo*, the nanosystem exploits the specific binding affinity of HA to CD44 receptors on macrophages to selectively accumulate at inflamed pancreatic sites. Subsequently, the cerium-based nanozyme efficiently scavenges ROS through the reversible redox cycling between Ce^3+^ and Ce^4+^, while the slow release of curcumin further suppresses the NF-κB signaling pathway and modulates inflammatory cytokine levels, thereby achieving synergistic anti-inflammatory and antioxidant effects. Collectively, these mechanisms substantially attenuate CP progression. This targeted ROS-scavenging and anti-inflammatory strategy holds promise as an alternative therapeutic approach for chronic pancreatitis.

## Graphical abstract

**Figure:**
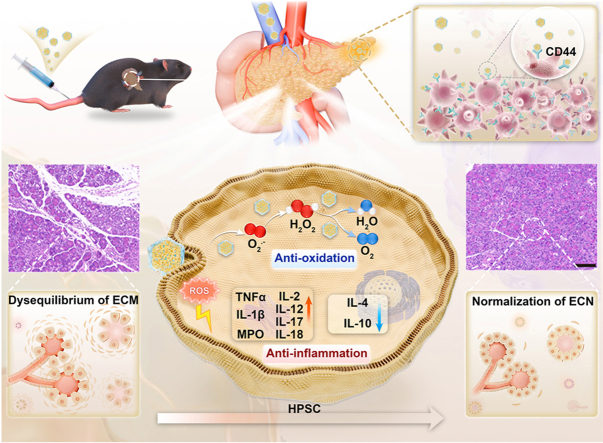

## 1 Introduction

Chronic pancreatitis (CP) is a lifelong progressive fibrosing inflammatory disease caused by the interplay of genetic and environmental factors [1]. Clinically, CP can lead to both exocrine and endocrine pancreatic insufficiency, accompanied by recurrent pain, severely impacting the patient's quality of life [2]. Moreover, it significantly increases the risk of pancreatic cancer [3]. The global prevalence of CP ranges from 13.5 to 52.4 cases per 100,000 people and has been steadily rising over the past five years, contributing to a substantial disease burden [2]. However, the current treatment for CP primarily involves palliative measures such as enzyme replacement and nutritional support, with no curative treatment available to date [4]. Therefore, the development of a safe and effective therapeutic strategy for CP is of critical importance for safeguarding human health.

Pancreatic fibrosis is a hallmark pathological feature of CP and represents the fundamental barrier to its cure [5]. Persistent and recurrent inflammatory stimuli are the primary drivers of fibrogenesis, with reactive oxygen species (ROS) serving as key mediators linking inflammation and fibrosis in the context of CP [6]. Under sustained inflammatory stress, excessive ROS are generated and accumulate in the pancreas through mechanisms such as mitochondrial dysfunction, abnormal activation of pancreatic enzymes, and respiratory bursts from immune cells [[6], [7], [8]]. These ROS directly induce oxidative damage in pancreatic cells and activate pro-inflammatory signaling pathways such as NF-κB, thereby establishing a vicious cycle between inflammation and oxidative stress [9,10]. Meanwhile, excessive ROS promote the activation of pancreatic stellate cells (PSCs) into fibrogenic myofibroblasts, which drive collagen deposition and pancreatic fibrosis, ultimately leading to irreversible tissue destruction [11]. Thus, ROS function as both amplifiers of inflammation and direct mediators of fibrosis, and targeting oxidative stress may offer a promising strategy for alleviating the pathological progression of CP. However, despite decades of investigation and numerous clinical studies, the therapeutic efficacy of antioxidant treatments in CP remains controversial [12,13]. Some studies have even questioned the clinical benefits of antioxidants in this setting [14]. This inconsistency may be attributed to the anatomical characteristics of the pancreas, which is located retroperitoneally and surrounded by vital organs and blood vessels, making it difficult for drugs to penetrate and directly reach the lesion site [15]. Moreover, although the pancreas is richly vascularized, its blood supply is unevenly distributed, potentially limiting the ability of therapeutic agents to achieve effective concentrations throughout the entire organ [16]. Therefore, developing an efficient drug delivery strategy to enhance the bioavailability of antioxidants may represent a viable solution to overcome these challenges. The NF-κB signaling pathway is widely recognized for its pivotal role in regulating immune and inflammatory responses, and it also plays a critical role in the pathogenesis of CP [17]. Huang et al. demonstrated that activation of NF-κB signaling in pancreatic acinar cells correlates with the severity of acute pancreatitis, and sustained activation can drive the progression toward chronic pancreatitis [18]. Moreover, Chen et al. reported that inhibition of NF-κB signaling attenuates the activation of PSCs and alleviates CP [19]. These findings suggest that simultaneously achieving efficient clearance of ROS and suppression of NF-κB signaling may yield enhanced therapeutic efficacy against CP.

Nanomaterials have emerged as a promising platform for drug delivery due to their small-sized particle size and unique structural and functional properties [[20], [21], [22]]. Representative nanomaterial systems—including lipid-based nanoparticles, polymeric nanoparticles, dendrimers, and inorganic nanoparticles—exhibit tunable physicochemical characteristics and enhanced targeting capabilities, which are particularly advantageous for biomedical applications [23,24]. Among these functional nanomaterials, antioxidant nanozymes—nanomaterials with intrinsic enzyme-mimicking activities—have attracted considerable attention in biomedical applications [[25], [26], [27], [28], [29]]. Cerium-based nanozymes, in particular, can selectively scavenge ROS by leveraging the reversible redox cycling between Ce^3+^ and Ce^4+^ oxidation states [30]. Notably, recent studies have reported the application of metal–organic framework (MOF)-based nanozymes in pancreatitis therapy. For instance, a Cu-centered MOF nanozyme with both SOD- and catalase-like activities was shown to alleviate acute pancreatitis by scavenging ROS and activating the PINK1/PARK2-mediated mitophagy pathway [31]. Cerium-based nanozymes derived from MOF exhibit additional advantages. Compared with other MOF nanozymes, cerium-based MOF nanozymes possess a smaller particle size and superior ROS-eliminating capacity, making them highly favorable for long-term antioxidant therapy in chronic inflammatory conditions [32]. Curcumin (Cur), a polyphenolic compound extracted from the rhizome of *Curcuma longa*, possesses well-documented anti-inflammatory and antioxidant properties [33]. Studies have demonstrated that Cur can exert anti-fibrotic effects in CP by inhibiting the activation of PSCs and suppressing the production of monocyte chemotactic protein-1 (MCP-1) induced by interleukin-1β (IL-1β) and tumor necrosis factor-α (TNF-α), thereby reducing the synthesis and secretion of extracellular matrix (ECM)-associated proteins [34,35]. These findings highlight the significant therapeutic potential of Cur in the treatment of CP. Hyaluronic acid (HA), an endogenous polysaccharide, binds with high affinity to the CD44 receptors that are markedly up-regulated on activated macrophages, thereby conferring nanomaterials with precise homing to sites of inflammation [[36], [37], [38]].

**Scheme 1:**
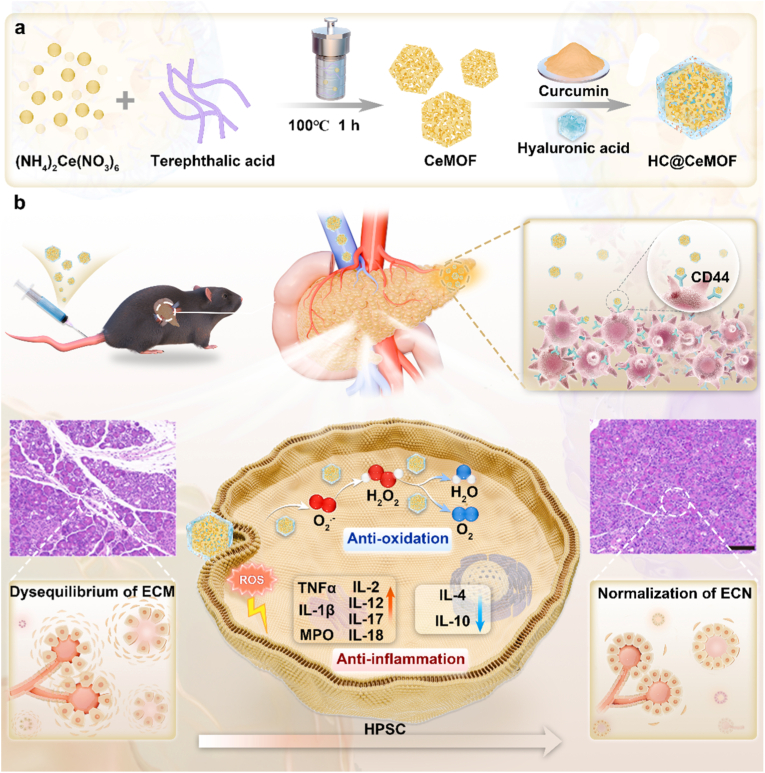
Schematic illustration of HC@CeMOF formation and therapeutic mechanism for chronic pancreatitis. (a) Ilustration of engineering HC@CeMOF synthesized using a hydrothermal synthesis method. (b) After entering the body, HC@CeMOF selectively accumulates in chronic pancreatitis lesions, where it inhibits the activation of pancreatic stellate cells through its antioxidant and anti-inflammatory effects, thereby reducing the progression of pancreatic fibrosis.

Here, we report the rational design of a Cur-loaded, small-sized cerium-based metal-organic framework (CeMOF) with intrinsic antioxidant activity, further coated with a HA shell to construct a targeted nanotherapeutic platform, termed HC@CeMOF, for the treatment of CP (Scheme 1). Leveraging the high expression of CD44 receptors on infiltrating macrophages within inflamed pancreatic tissues, the HA corona facilitates the preferential accumulation of HC@CeMOF at sites of inflammation. Once localized, HC@CeMOF exerts synergistic therapeutic effects through efficient scavenging of ROS by CeMOF and potent anti-inflammatory and anti-fibrotic actions of Cur. This synergistic mechanism markedly modulates the inflammatory cytokine profiles and suppresses NF-κB signaling, thereby ameliorating CP progression.

## 2 Methods

### 2.1 Materials and characterizations

HA (molecular weight: >1.8 MDa, H293496), Cerium Diammonium Nitrate (H_8_CeN_8_O_18_, A105050), Cur (C_21_H_20_O_6_, C140600), 1,1-Diphenyl-2-picrylhydrazyl free radical (DPPH·) (D273092), 2,2′-Azino-di-(3-ethylbenzothiazoline)-6-sulfonic acid (ABTS·^+^) (A276045), and 2-phenyl-4,4,5,5-tetramethylimidazoline-1-oxyl-3-oxide (PTIO·) (P160514) were purchased from Aladdin Reagent Co., Ltd. (Shanghai, China). N, N-Dimethylformamide (C_3_H_7_NO, 01375912) and terephthalic acid (C_8_H_6_O_4_, 01000167) were obtained from Shanghai Titan Technology Co., LTD. Cell Counting Kit-8 (CCK 8, CK04), (11184ES08), Annexin V-FITC/PI Apoptosis Detection Kit (40302ES50), and Live/Dead cell staining kit (40747ES76) were purchased from Yeasen Biotechnology (Shanghai, China) Co. Ltd. α-Smooth Muscle Actin (D4K9N) XP® Rabbit mAb (α-SMA, 19245S), NF-κB p65 (D14E12) XP® Rabbit mAb (8242), Phospho-NF-κB p65 (Ser536) (93H1) Rabbit mAb (3033), and COL1A1 (E8F4L) XP® Rabbit mAb (COL-1, 72026S) were bought from Cell Signaling Technology. Transmission electron microscopy (TEM) was utilized to examine the morphological characteristics of the nanozymes using an JEM2100F microscope (Japan, 200 kV). A field emission scanning electron microscope (ZEISS Sigma 300, Germany) was utilized for scanning electron microscopy (SEM) imaging. X-ray diffraction (XRD, Rigaku SmartLab SE, Japan) was performed to observe the crystal structure of the samples. X-ray photoelectron spectroscopy (XPS) was conducted using a Thermo Fisher Scientific K-Alpha (USA) to K-Alpha (USA) to analyze the valence state of the elements. The chemical structure of the samples was analyzed using Fourier transform infrared (FTIR) spectroscopy with a Thermo Fisher Scientific Nicolet iS20 instrument (USA). The nitrogen adsorption isotherms were measured using a surface area and porosity analyzer (Micromeritics ASAP 2460). Human pancreatic stellate cells (HPSCs) and RAW264.7 cells were purchased from the Cell Bank of the Chinese Academy of Sciences (Beijing, China). Cells were cultured in DMEM/F12 supplemented with 10 % FBS and 1 % penicillin/streptomycin under standard conditions (5 % CO_2_, 37 °C). All animal procedures in this study were approved by the Ethics Committee of the First Affiliated Hospital of Nanchang University (Nanchang, Jiangxi Province, China, Approval Number: CDYFY-IACUC-202505GR063). C57BL/6 male mice (5–6 weeks old, weighing 18–25 g) were obtained from Jiangsu GemPharmatech LLC and were housed in the animal room of the Animal Laboratory, Science and Technology Department, The First Affiliated Hospital of Nanchang University. They were maintained in a specific pathogen-free (SPF) environment under a 12-h light-dark cycle and were provided a standard diet adlibtum.

### 2.2 Synthesis of HC@CeMOF

Using a hydrothermal synthesis method and by adjusting the ratio of H_2_BDC to (NH_4_)_2_Ce(NO_3_)_6_, we successfully prepared small-sized Ce-MOF nanozymes. Specifically, 0.354 g of H_2_BDC was dissolved in 24 mL of DMF, followed by the addition of 8 mL of an aqueous solution containing 1.16 g of (NH_4_)_2_Ce(NO_3_)_6_ at room temperature. The mixture was stirred for 10 min and then transferred into a Teflon-lined autoclave, where it was heated at 100 °C for 1 h. After naturally cooling to room temperature, the resulting precipitate was collected and dialyzed against deionized water using a dialysis membrane with a molecular weight cutoff of 14,000 Da for 2 days. The dialyzed solution was then lyophilized for storage. Using the above method, 0.56 g of CeMOF can be obtained, with a yield of approximately 87 %. For drug loading, 5 mg of Cur was dissolved in 1 mL of DMSO and added dropwise into a CeMOF solution (2.5 mg/mL), followed by overnight stirring. The mixture was centrifuged, and the pellet was freeze-dried to obtain Cur@CeMOF nanozymes. Finally, 1 mg/mL HA solution was added to the Cur@CeMOF suspension, stirred for 24 h, and then centrifuged and lyophilized to obtain the final HA-modified Cur@CeMOF formulation, named HC@CeMOF. We synthesized three independent batches of the material at different time points, and for all subsequent experiments requiring replication, we performed the assays three times using each of these independent batches.

### 2.3 Thermogravimetric analysis (TGA)

TGA was performed using a simultaneous thermal analyzer (NETZSCH, STA449F3). Specifically, freeze-dried samples were placed in alumina crucibles and equilibrated at 30 °C, then heated to 800 °C at a rate of 10 K/min, with the mass loss recorded throughout the heating process. All measurements were conducted under an inert atmosphere using nitrogen at a flow rate of 40 mL/min. Empty alumina crucibles served as the reference in all experiments. The instrument was calibrated, and baseline correction was performed before testing each sample using an alumina crucible on the built-in balance. OriginLab software was used for plotting and analyzing the resulting thermogravimetric curves.

### 2.4 The load capacity of HC@CeMOF and *in vitro* drug release

Cur solutions of different concentrations were mixed and stirred with the CeMOF solution, followed by centrifugation to collect the supernatant. The absorbance of the supernatant at 430 nm was measured using a UV–vis spectrophotometer, and the concentration of free Cur was determined based on a standard calibration curve. The encapsulation efficiency and drug loading were then calculated accordingly.

$$\text{Encapsulation}\;\text{efficiency}=\frac{m_{load}}{m_{0}}\times 100\%$$

$$\text{Drug}\;\text{loading}\;\text{capacity}=\frac{m_{load}}{m+m_{0}}\times 100\%$$

where m_load_ is the amount of Cur successfully loaded, m_0_ is the initial amount of Cur added, and m is the mass of CeMOF.

The *in vitro* drug release rate was determined based on established methods from previous literature [39]. In brief, Cur (5 mg) was first dissolved in a small volume of DMSO (1 mL) to ensure complete solubilization. Then, Cur solutions of varying concentrations (1, 2, 3, 4, and 5 μg/mL) were prepared. The absorbance of the supernatants was recorded at a wavelength of 430 nm using a UV–Vis–NIR spectrophotometer (UV1800PC, China), and a standard calibration curve of Cur was subsequently constructed. Then, 3 mL of the HC@CeMOF solution was placed into a dialysis bag with a molecular weight cutoff of 3500 Da, which was subsequently immersed in a 50 mL centrifuge tube containing 10 mL of PBS. The tube was incubated in a water bath shaker at 37 °C (60 rpm). At 0, 2, 4, 6, 8, and 10 h, 1 mL of the release medium was withdrawn and replaced with 1 mL of fresh PBS. The collected release medium (1 mL) was directly analyzed by UV–Vis–NIR spectrophotometry at 430 nm, and the concentration of Cur released from the HC@CeMOF nanozymes at each time point was calculated based on the standard curve. Similarly, equal amounts of HC@CeMOF were placed in PBS solutions at pH 6.5, containing hyaluronidase (10 U/mL) or hydrogen peroxide (10 mM), and incubated at 37 °C. Cur release was measured at designated time points.

### 2.5 Evaluation of free radical-scavenging capacity

The scavenging capacity of HC@CeMOF toward reactive nitrogen species (RNS) and ROS was systematically evaluated using DPPH·, PTIO·, and ABTS·^+^ scavenging assay. For the DPPH· assay, various amounts of HC@CeMOF (40 μg, 80 μg, 120 μg, and 160 μg) were added to 2 mL of DPPH· solution. In the PTIO· and ABTS·^+^ assay, the same quantities of HC@CeMOF were mixed with 2 mL of PTIO· and ABTS·^+^ solution. All mixtures were incubated in the dark at 37 °C for 30 min, and the absorbance was measured at 519 nm, 557 nm and 734 nm, respectively, using a UV spectrophotometer. The radical scavenging rate was calculated using the following formula:

$$Scavenging\;ratio=\frac{A0-Ab}{A0}\times 100\%$$

In this equation, A0 is the absorbance of the blank group, and Ab is the absorbance of the supernatant of experimental groups.

Meanwhile, the H_2_O_2_ and O_2_^−^ scavenging activities of the nanomaterials were evaluated using hydrogen peroxide (Beyotime, S0038) and superoxide assay kits (Beyotime, S0060). Specifically, 2 mL of hydrogen peroxide solution (10 mM) was incubated with an equal amount of HC@CeMOF at 37 °C in the dark for 15 min, and the absorbance at 240 nm was measured using a UV–vis spectrophotometer. Superoxide scavenging was assessed according to the kit instructions: 8, 16, 24, and 32 μg of HC@CeMOF were added to 400 μL of working solution, incubated at 37 °C in the dark for 30 min, and the absorbance of the supernatant was measured at 450 nm.

### 2.6 The determination of Michaelis constant (K_m_) and maximum reaction rate (V_max_)

The HC@CeMOF (60 μg/mL) was mixed with different concentrations of hydrogen peroxide in 400 μL of 10 mM phosphate-buffered saline (PBS, pH 7.5). The absorbance at 240 nm was recorded every 5 s to determine the initial reaction rate. Kinetic parameters for H_2_O_2_ decomposition (V_max_, nM·s^−1^; K_m_, mM) were calculated using a Lineweaver–Burk plot.

$$\frac{1}{V}=\frac{K_{m}}{V_{\max}}\times \frac{1}{\left[S\right]}+\frac{1}{V_{\max}}$$

In this equation, [S] represents the substrate concentration, V is the apparent initial reaction rate, V_max_ denotes the maximum reaction rate, and K_m_ is the Michaelis constant.

### 2.7 Cell compatibility

This study systematically evaluated the cytocompatibility of the nanozymes using three complementary methods: flow cytometry for apoptosis detection, CCK-8 assay for cell proliferation and viability analysis, and Live/Dead Cell Staining assay. The specific procedures are as follows:

***Apoptosis analysis by flow cytometry***: HPSCs were seeded in six-well plates at a density of 1 × 10^6^ cells per well. After overnight incubation to allow cell attachment, the culture medium was replaced with fresh medium containing various concentrations of HC@CeMOF. Following a 24 h treatment period, the cells were harvested and gently washed twice with PBS to remove residual compounds. The cell pellets were then resuspended in an appropriate binding buffer and incubated in the dark with 5 μL of Annexin V-FITC for 15 min. Subsequently, 10 μL of propidium iodide was added, and incubation continued for another 5 min. Finally, apoptotic cells were quantitatively analyzed using a flow cytometer (Agilent, NovoCyte 3110, USA).

***CCK-8 assays***: HPSCs were seeded into 96-well plates at a density of 1 × 10^5^ cells per well. Once cell adherence was established, the culture medium was replaced with 100 μL of fresh medium containing varying concentrations of HC@CeMOF, followed by incubation for 24, 48, or 72 h. After the designated incubation period, 100 μL of a 10 % CCK-8 reagent working solution was added to each well and incubated at 37 °C for 30 min. The absorbance of the water-soluble formazan product was measured at 450 nm using a microplate reader (SpectraMax® i3, Molecular Devices, USA). Each experimental group included three replicate wells, and cell viability was calculated using the following formula:

$$Cell\;viability\;\left(\%\right)=\frac{OD_{experimental\;groups}-{OD}_{blank\;groups}}{{OD}_{control\;groups}-{OD}_{blank\;groups}}\times 100\%$$

***Live/Dead Cell Staining assay***: Following the CCK-8 assay treatment, HPSCs were incubated with calcein-AM and propidium iodide (PI) dyes added directly to the culture medium. The cells were then maintained at 37 °C for 30 min in the dark. Subsequently, fluorescence imaging was performed using an inverted fluorescence microscope (Leica DMIL LED, Germany). Quantitative analysis of fluorescence intensity using a three-dimensional fluorescence topographic map (ImageJ software 1.8.0_322, http://imagej.nih.gov/ij).

### 2.8 Hemolysis assay

The hemolysis assay of the HC@CeMOF was performed following previously reported protocols [40]. Briefly, fresh whole blood was collected from healthy rats and centrifuged at 3000 rpm for 15 min to isolate the cellular components. The harvested erythrocytes were then diluted with normal saline. A total of 300 μL of the diluted red blood cell suspension was transferred into individual test tubes. For the positive control, 1.2 mL of deionized water was added; for the negative control, 1.2 mL of PBS was used. Experimental groups received 1.2 mL of saline solutions containing varying concentrations of HC@CeMOF. After incubation at 37 °C for 2 h, the samples were centrifuged and the absorbance of the supernatant was measured at 540 nm using a microplate reader. The hemolysis rate was calculated using the following formula:

$$Hemolysis\;rate\;\left(\%\right)=\frac{{OD}_{experimental\;groups}-{OD}_{negative\;control\;group}}{{OD}_{positive\;control\;group}-{OD}_{negative\;control\;group}}\times 100\%$$

### 2.9 Evaluation of intracellular free radical-scavenging capacity

RAW264.7 cells were seeded in 6-well plates at a density of 5 × 10^5^ cells per well and incubated for 24 h. Subsequently, the culture medium was replaced with one of the following: fresh medium (control group), fresh medium containing H_2_O_2_, medium containing Cur and H_2_O_2_, medium containing Cur@CeMOF and H_2_O_2_, or medium containing HC@CeMOF and H_2_O_2_. Cells were then incubated for an additional 6 h. After treatment, cells were incubated with the ROS-sensitive fluorescent probe 2′,7′-dichlorofluorescin diacetate (DCFH-DA) (S0033, Beyotime Biotechnology, China) for 20 min. The cells were then washed three times with PBS by centrifugation. Intracellular ROS levels were observed using an inverted fluorescence microscope and quantitatively analyzed by flow cytometry. The excitation wavelength was set to 488 nm, and the emission wavelength was set to 525 nm. Quantitative analysis of fluorescence intensity using a three-dimensional fluorescence topographic map (ImageJ software 1.8.0_322, http://imagej.nih.gov/ij).

### 2.10 Evaluation of the *in vitro* anti-fibrotic ability of nanozymes

Cur, Cur@CeMOF, and HC@CeMOF were co-incubated with cells for 24 h, respectively, followed by evaluation of the *in vitro* anti-fibrotic effects of the nanozymes using Western blotting and immunofluorescence assays. The detailed procedures are as follows:

***Western blotting*:** Cell lysis was performed using RIPA buffer (Beyotime Biotechnology, China), and the resulting whole-cell lysates were centrifuged for purification. Protein samples were separated by 10 % SDS-PAGE and subsequently transferred onto PVDF membranes (Merck Millipore, Darmstadt, Germany). After blocking with 5 % non-fat milk at room temperature for 1 h, the membranes were incubated overnight at 4 °C with the corresponding primary antibodies. The next day, membranes were further incubated with horseradish peroxidase (HRP)-conjugated secondary antibodies (Epizyme Biotech, China, LF102) at room temperature for 1.5 h. Protein bands were visualized using an ECL detection system (Amersham Imager 600, GE). GAPDH (Epizyme Biotech, China, LF206) was used as the internal control. Details of the primary antibodies used in this study are provided in Section 2.1.

***Immunofluorescence staining in vitro***: After treatment with different formulations, HPSCs were washed and fixed *in vitro* using 4 % paraformaldehyde, followed by rinsing with Hank's Balanced Salt Solution. Non-specific binding sites were blocked with 5 % donkey serum, after which cells were incubated overnight at 4 °C with rabbit anti-human α-SMA or COL-1 primary antibodies. Subsequently, cells were washed and incubated in the dark with a goat anti-rabbit secondary antibody (Abcam, 150080). Nuclear counterstaining was performed using DAPI (Thermo Fisher, P36981). Finally, cellular fluorescence was evaluated using confocal laser scanning microscopy (CLSM, ZEISS LSM880, Germany), and fluorescence intensity was quantified using ImageJ software.

### 2.11 Molecular docking and molecular dynamics

A semi-flexible docking strategy was employed to evaluate the binding affinity and binding conformation between HA and the CD44 protein. The CD44 structure was retrieved from the Protein Data Bank and preprocessed using PyMOL. Hydrogen atoms were added and partial charges were calculated for both the ligand and the protein using AutoDock Vina. The active binding site was identified based on the ligand-receptor complex information available in the PDB database. Molecular docking was performed with AutoDock Vina, and binding affinity was assessed based on docking scores (binding energies). The docking poses and interactions were further visualized and analyzed using Discovery Studio Visualizer to elucidate the binding mode. To better simulate the interaction between HA and CD44 under physiological conditions, molecular dynamics simulations were also conducted. Molecular dynamics simulations were performed using GROMACS 2022. Hydrogen atoms were added to the protein via the pdb2gmx module, followed by system preparation with editconf, solvate, and grompp, including box definition (5.979 × 6.599 × 7.250 nm^3^), solvent addition, and charge neutralization with counter-ions. Small molecule charges were assigned using the AM1-BCC method in Antechamber, with GAFF for ligands, Amber ff99SB for proteins, and TIP3P water model. The system underwent 50,000 steps of energy minimization using the steepest descent method, followed by 100 ps equilibration under NVT and NPT ensembles with position restraints on heavy protein atoms. A 100 ns production run was then conducted at 310 K and 1 bar using the Parrinello-Rahman barostat. Long-range electrostatics were calculated using the Particle-Mesh Ewald method, and a 1 nm cutoff was applied to van der Waals interactions. The SHAKE algorithm constrained bonds involving hydrogen atoms. Trajectory data were recorded every 10 ps. These experiments were performed by Shanghai Huiyan Zhiyao Biotechnology Co., Ltd (China).

### 2.12 Animal studies

***In vivo* biosafety**: The *in vivo* biosafety of the nanozymes was evaluated first. In brief, C57BL/6 mice of similar body weight were randomly divided into a control group and an experimental group (n = 3). The experimental groups received tail vein injections of HC@CeMOF (6 mg/kg/day), while the control group was administered an equivalent volume of normal saline. All mice were allowed free access to food, and their body weight changes were monitored. On days 1, 7, 14, and 28, the mice were euthanized, and blood samples were collected for serum biochemical and hematological analyses. Additionally, major organs-including the heart, liver, spleen, lungs, and kidneys-were harvested and subjected to hematoxylin and eosin (H&E) staining to assess systemic toxicity and overall biosafety.

***Animal model***: A caerulein-induced CP model was established by repeated induction of acute pancreatitis. Specifically, mice were randomly assigned to the control group, CP model group, Cur group, Cur@CeMOF group, and HC@CeMOF group. The control group received 200 μL of normal saline via tail vein injection, while the other groups were administered caerulein intraperitoneally at a dose of 50 μg/kg per hour based on body weight, for 6 consecutive injections per day, 3 days per week, over a period of 4 weeks. Body weights were recorded after the final injection each week, and animals were euthanized following the last injection. Starting from the second week of caerulein administration [17], the CP model group, Cur group, Cur@CeMOF group, and HC@CeMOF group were treated via daily tail vein injections for 3 consecutive weeks with normal saline, Cur (6 mg/kg/day), Cur@CeMOF (6 mg/kg/day), or HC@CeMOF (6 mg/kg/day), respectively. The specific method for preparing the Cur solution is as follows: Cur was first dissolved in DMSO to prepare a stock solution, then diluted to a nontoxic therapeutic concentration with a PEG-400/sterile saline mixture. Specifically, 10 mg Cur in 1 mL DMSO was prepared, and 30 μL of this solution was mixed with 2 mL PEG-400 and 3 mL saline to a final volume of 5 mL.

***Evaluation of the in vivo targeting ability of HC@CeMOF***: After establishing the CP model using the aforementioned method, mice were administered a tail vein injection of Cy5.5-labeled HC@CeMOF. The mice were euthanized at 5 min, 30 min, 1 h, 6 h, 12 h, and 24 h post-injection, and fluorescence intensity in the pancreatic tissue was measured using an IVIS imaging system (Tanon ABL X6, China). To further assess the biodistribution of the nanomaterial following systemic administration, nine CP model mice and three healthy control mice were randomly selected. The CP model mice received intravenous injections of equal volumes of normal saline, Cy5.5-labeled Cur@CeMOF, or Cy5.5-labeled HC@CeMOF, respectively. The healthy mice were administered Cy5.5-labeled HC@CeMOF via tail vein injection. 2 h after injection, all mice were euthanized, and fluorescence intensity in the heart, liver, spleen, lungs, kidneys, and pancreas was analyzed using the small animal imaging system. In addition, the Ce content in each organ was quantified using inductively coupled plasma (ICP) analysis (iCAP 7000 SERIES, Thermo Fisher Scientific, USA). Selected pancreatic tissues were sectioned using a cryostat for staining and subsequently observed under an upright fluorescence microscope. Moreover, to further investigate the targeting role of HA, we pre-injected free HA (20 mg/kg) or saline 30 min before administering Cy5.5-labeled HC@CeMOF to saturate CD44 receptors. The HC@CeMOF accumulation in both groups was evaluated using IVIS imaging, and the pancreatic Ce content was also quantified using ICP analysis.

***Evaluation of the in vivo therapeutic effect of HC@CeMOF on CP***: After 4 weeks of caerulein-induced CP modeling and 3 weeks of nanozyme treatment, the mice were euthanized, and serum samples were collected. The expression levels of inflammatory cytokines (including IL-1β, IL-2, IL-4, IL-10, IL-12, IL-17, IL-18, MPO activity, and TGF-α) were measured using enzyme-linked immunosorbent assay (ELISA) kits according to the manufacturer's instructions (FAM-INF-1-96, RayBiotech, Inc., Guangzhou, China). The pancreatic tissues were excised, photographed, and weighed, and the pancreas-to-body weight ratio was recorded. A portion of the pancreatic tissue was used for Western blot (WB) assess to detect the expression of fibrosis-related proteins and proteins associated with the NF-κB signaling pathway according to the method previously described [5]. The remaining pancreatic tissue was embedded and processed for histological evaluation, including H&E staining, Masson staining, Sirius Red staining, immunofluorescence staining, and immunohistochemistry, to assess the degree of pancreatic fibrosis in the mice. These pathological experiments were performed by Wuhan servicebio technology Co., Ltd (China). In addition, we monitored serum pancreas-related indices in each group after treatment. Briefly, mice were fasted overnight from 8:00 a.m. to 8:00 p.m. and then euthanized. Blood samples were collected and centrifuged at 3000 rpm for 15 min, and the supernatant was obtained as serum. Serum lipase and amylase levels were measured using a lipase assay kit (Nanjing Jiancheng Bioengineering Institute, A054-2-1) and an amylase assay kit (Nanjing Jiancheng Bioengineering Institute, C016-1-2).

### 2.13 Statistical analysis

All the data are presented as the mean ± standard deviation (SD). Statistical analysis was performed using IBM SPSS Statistics 25.0 (IBM Corp, Chicago, IL, USA). Graphs were generated using GraphPad Prism 9.5 (GraphPad Software, La Jolla, CA, USA) and OriginLab 9.0 (OriginLab Corporation, Northampton, MA, USA). Differences between two groups were compared using Student's t-test, while differences among three or more groups were compared using one-way analysis of variance (ANOVA). A p-value <0.05 was considered to indicate statistical significance (ns: not significant, ∗*p* < 0.05, ∗∗*p* < 0.01, and ∗∗∗*p* < 0.001).

## 3 Results and discussion

### 3.1 Preparation and structure of HC@CeMOF

**Fig. 1:**
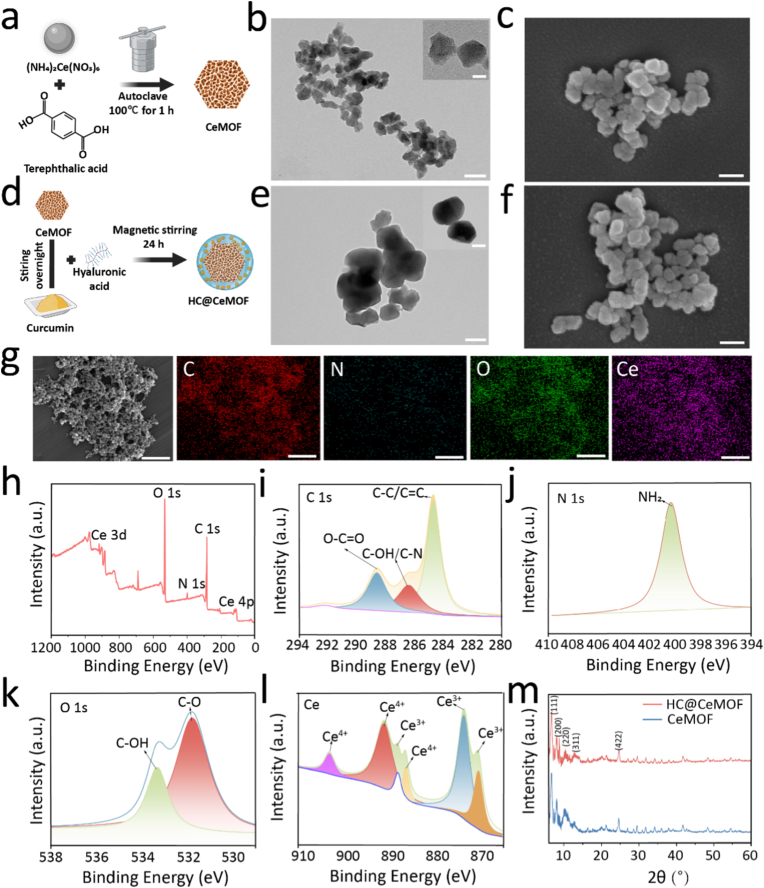
The synthesis and structure of CeMOF and HC@CeMOF. (a) The illustration of CeMOF preparation. (b) TEM images of CeMOF, bars represent 100 nm; Enlarged TEM: bars represent 20 nm. (c) SEM images of CeMOF. Bars represent 100 nm. (d) The illustration of HC@CeMOF preparation. (e) TEM images of HC@CeMOF, bars represent 100 nm; Enlarged TEM: bars represent 20 nm. (f) SEM images of HC@CeMOF. Bars represent 100 nm. (g) EDS mapping of the distribution of C, N, O, and Ce on CeMOF, bar represent 500 nm. (h) XPS of CeMOF. High-resolution XPS spectra of CeMOF, including (i) C 1s spectrum fitted into C–C/C=C, C–OH/C–N and O=C–O components, (j) N 1s spectrum displaying an NH_2_-associated peak, (k) O 1s spectrum deconvoluted into C–OH and C=O species, and (l) Ce 3d spectrum revealing mixed Ce^3+^/Ce^4+^ states. (m) XRD of CeMOF and HC@CeMOF.

Herein, we synthesized small-sized CeMOF nanoparticles via a one-step hydrothermal method by regulating the precursor concentrations, following procedures previously described in the literature [32] (Fig. 1a). Specifically, ammonium cerium nitrate and terephthalic acid were heated at 100 °C for 1 h in a reaction vessel. The high-valence metal ion Ce^4+^, with strong coordination capability, formed coordination equilibria with the carboxyl groups of terephthalic acid, resulting in the self-assembly of a topologically structured MOF network. TEM images revealed that the synthesized CeMOF nanoparticles exhibited a polyhedral cubic morphology with an average size of approximately 60 nm (Fig. 1b), which was consistent with the results observed by SEM (Fig. 1c).

Next, we utilized electrostatic attraction to load negatively charged Cur onto CeMOF, and subsequently employed a simple physical stirring method to enable the rapid encapsulation of the particles with HA, yielding small-sized nanozyme particles, denoted as HC@CeMOF (Fig. 1d). Here, we loaded Cur onto CeMOF using different Cur concentrations and found that a concentration of 2 mg/mL produced the highest encapsulation efficiency and loading capacity (Figure S1). Therefore, we used 2 mg/mL Cur as the final concentration for preparing HC@CeMOF. TEM analysis showed that, compared to bare CeMOF, HC@CeMOF exhibited an additional organic coating on the surface (Fig. 1e). SEM images also demonstrated a slight increase in particle size after HA encapsulation (Fig. 1f). Elemental mapping of HC@CeMOF confirmed the uniform distribution of C, N, O, and Ce elements, with nitrogen mainly originating from HA, indicating successful coating with both Cur and HA (Fig. 1g). To further clarify HA's effect, the FTIR spectrum of HA@MOF (Figure S2) was analyzed. Hydroxyl signals suggest possible interactions, indicating HA coats the MOF surface via hydrogen bonding.

We further examined the oxidation states of cerium in the CeMOF nanoparticles. XPS analysis revealed that the primary elements in the material were carbon (C), nitrogen (N), oxygen (O), and cerium (Ce) (Fig. 1h). The C 1s spectrum features a main peak at 284.8 eV assigned to C–C/C=C bonds, along with peaks at 286.5 eV and 288.7 eV corresponding to C–O/C–N and O–C=O species, respectively, confirming the presence of polar functional groups and surface carboxylates (Fig. 1i). The N 1s spectrum shows a single peak at 400.4 eV characteristic of –NH_2_/–NH– groups (Fig. 1j). In the O 1s spectrum (Fig. 1k), the peak at 529.5 eV originates from lattice oxygen in Ce–O–Ce units, whereas the 531.5 eV component is attributed to defect-related or hydroxyl oxygen (Ce–OH/Ce–O–C), indicating abundant surface hydroxyls and oxygen vacancies. The peaks at 533.1 eV and 534.8 eV arise from carboxylate oxygen and weakly adsorbed species such as H_2_O or CO_2_. These features collectively demonstrate the coexistence of lattice oxygen and reactive surface oxygen species that can serve as active sites. The Ce 3d spectrum exhibits typical spin–orbit–split multiplets (Fig. 1l); peaks at 886.3 eV and 901.8 eV correspond to Ce^3+^ (3d_5_⁄_2_/3d_3_⁄_2_), while features at 882.9, 899.8, 905.0, and the satellite at 917.6 eV are assigned to Ce^4+^. The simultaneous presence of Ce^3+^ and Ce^4+^ confirms a mixed-valence state, which likely originates from mild reduction during hydrothermal synthesis and the formation of intrinsic oxygen vacancies [32,41]. Such valence fluctuations endow the material with excellent oxygen mobility and surface redox activity, both of which are crucial for subsequent catalytic reactions and radical-scavenging processes [42,43].

Furthermore, XRD analysis was conducted to characterize the crystalline phases of CeMOF and HC@CeMOF (Fig. 1m). The results show that the synthesized CeMOF exhibits a cubic crystalline structure consistent with the diffraction pattern of Ce-UIO-66-BDC [32]. For HC@Ce-MOF, only a slight decrease in peak intensity was observed, with no noticeable peak shifts or additional diffraction peaks. This indicates that HA coating does not alter the structural framework of Ce-MOF, confirming the successful synthesis. Compared to uncoated CeMOF, HC@CeMOF exhibited slightly decreased overall diffraction intensity, likely due to the amorphous nature of the Cur and HA coating, which partially masked the diffraction signals. Nonetheless, the characteristic peaks of CeMOF were preserved, suggesting that the framework structure remained intact. We further examined the surface area and porosity of CeMOF and HC@CeMOF. Due to HA coating and Cur occupying the micropores, the composite showed a clear reduction in surface area, decreasing from 1573.4 m^2^/g to 875.7 m^2^/g (Figure S3). This confirms that the added polymer and drug molecules reduced the nitrogen-accessible pore volume. Taken together, these results confirmed the successful generation of HC@CeMOF nanoparticles.

### 3.2 Characterizations of HC@CeMOF

**Fig. 2:**
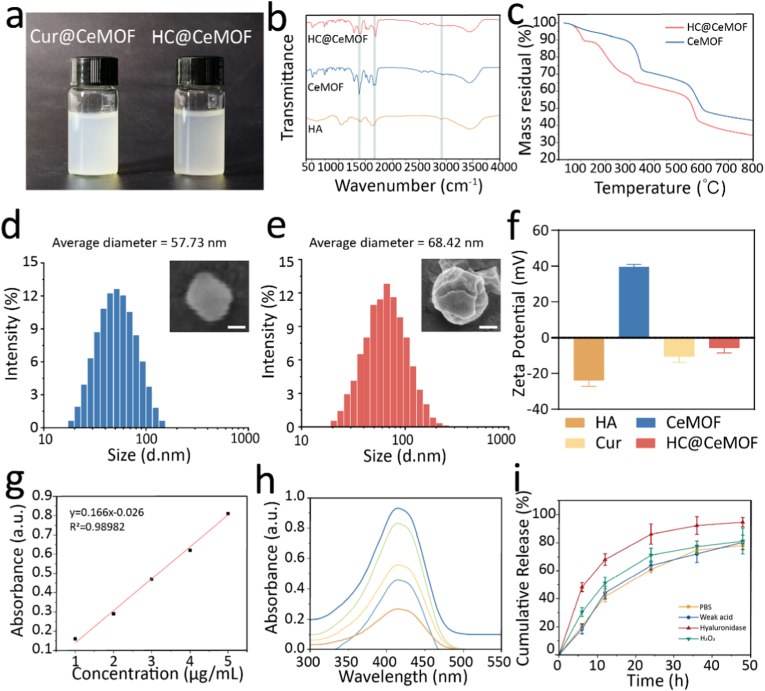
The characterizations of CeMOF and HC@CeMOF. (a) Optical images of CeMOF and HC@CeMOF. (b) FTIR of CeMOF and HC@CeMOF. (c) TG of CeMOF and HC@CeMOF. (d) The particle size distribution of CeMOF, bar represent 20 nm. (e) The particle size distribution of HC@CeMOF, bar represent 20 nm. (f) Zeta potential of HA, Cur, CeMOF and HC@CeMOF. (g) The standard curve of Cur. (h) The ultraviolet absorption spectrum of Cur. (i) *In vitro* release rate of Cur in HC@CeMOF under different conditions. Data are presented as the mean ± SD (n = 3).

Based on the optical images, both Cur@CeMOF and HC@CeMOF appear as milky white solutions that remain well-dispersed in water without precipitation at room temperature (Fig. 2a). FTIR was employed for further material characterization (Fig. 2b). Absorption bands at 1641–1554 cm^−1^ and 1435–1373 cm^−1^ can be primarily attributed to the asymmetric and symmetric stretching vibrations of the carboxylate group (COO^−^), respectively. These bands were consistently observed across all three samples, indicating the presence of similar surface functional groups. Notably, HC@CeMOF exhibits an additional absorption band between 1600 and 1500 cm^−1^, attributed to aromatic C=C skeletal vibrations, representing the characteristic fingerprint of Cur. The absence of significant peak shifts before and after compositing indicates that the MOF framework remains intact, while the presence of Cur-specific peaks confirms its successful incorporation. Fig. 2c shows the TGA of CeMOF and HC@CeMOF under a nitrogen atmosphere. HC@CeMOF exhibits a more rapid mass loss below 200 °C, attributed to the presence of low-temperature decomposable components from HA and Cur. Major decomposition between 200 and 350 °C corresponds to the breakdown of benzoic acid, HA, and Cur. Further decomposition of the framework occurs between 500 and 600 °C, leaving behind CeO_2_ as the final residue. Compared to CeMOF, HC@CeMOF demonstrates a lower residual mass (36 %) due to the incorporation of Cur and HA.

Subsequently, we assessed the hydrated particle size of CeMOF (Fig. 2d) and HC@CeMOF (Fig. 2e). The size distribution follows a normal distribution, with average diameters of 57.73 nm (PDI = 0.141) and 68.42 nm (PDI = 0.112), respectively. Fig. 2f displays the Zeta potential of each component. Due to electrostatic interactions (HA: −23.93 mV, CeMOF: 39.53 mV, Cur: −10.56 mV), HA and Cur can spontaneously bind to CeMOF, resulting in a final Zeta potential of approximately −5.73 mV for HC@CeMOF. To evaluate the stability of nanomaterials stability, dynamic light scattering (DLS) and Zeta potential measurements were conducted over 14 days at room temperature. The particle size of CeMOF increased by only ∼3 nm after 14 days, which is negligible. Moreover, its Zeta potential remained stable at approximately 39 mV (Figure S4). Similarly, HC@CeMOF also showed minimal change in size and Zeta potential throughout the study (Figure S5). These results indicate that both CeMOF and HC@CeMOF possess excellent stability, maintaining their physical properties for at least 14 days under ambient conditions. This also provides a theoretical basis supporting their ability to remain stable and exert sustained effects at the target site. The Cur loading capacity is critical for evaluating the therapeutic efficacy of HC@CeMOF. Using UV–Vis spectrophotometry, Cur showed a maximum absorbance at 420 nm. A standard calibration curve was constructed based on this wavelength (Fig. 2g and h). Furthermore, the cumulative release of Cur from HC@CeMOF reached 78 % within 48 h in water (Fig. 2i). The results also showed that Cur release from HC@CeMOF varied significantly under different conditions. Hyaluronidase accelerated the degradation of surface HA, exposing more pores and promoting faster Cur release, reaching 48.2 % at 6 h. Release in hydrogen peroxide was slightly faster than in PBS or mildly acidic conditions, likely because oxygen generated during H_2_O_2_ decomposition provided additional driving force, enhancing Cur release (Fig. 2i).

Collectively, these findings confirm that the HC@CeMOF nanoparticles constructed in this study possess small-sized particle size, high drug-loading capacity, excellent colloidal stability, and sustained-release properties, all of which provide a solid foundation and significant potential for the treatment of chronic pancreatitis.

### 3.3 *In vitro* cytotoxicity evaluation and ROS scavenging activity of HC@CeMOF

**Fig. 3:**
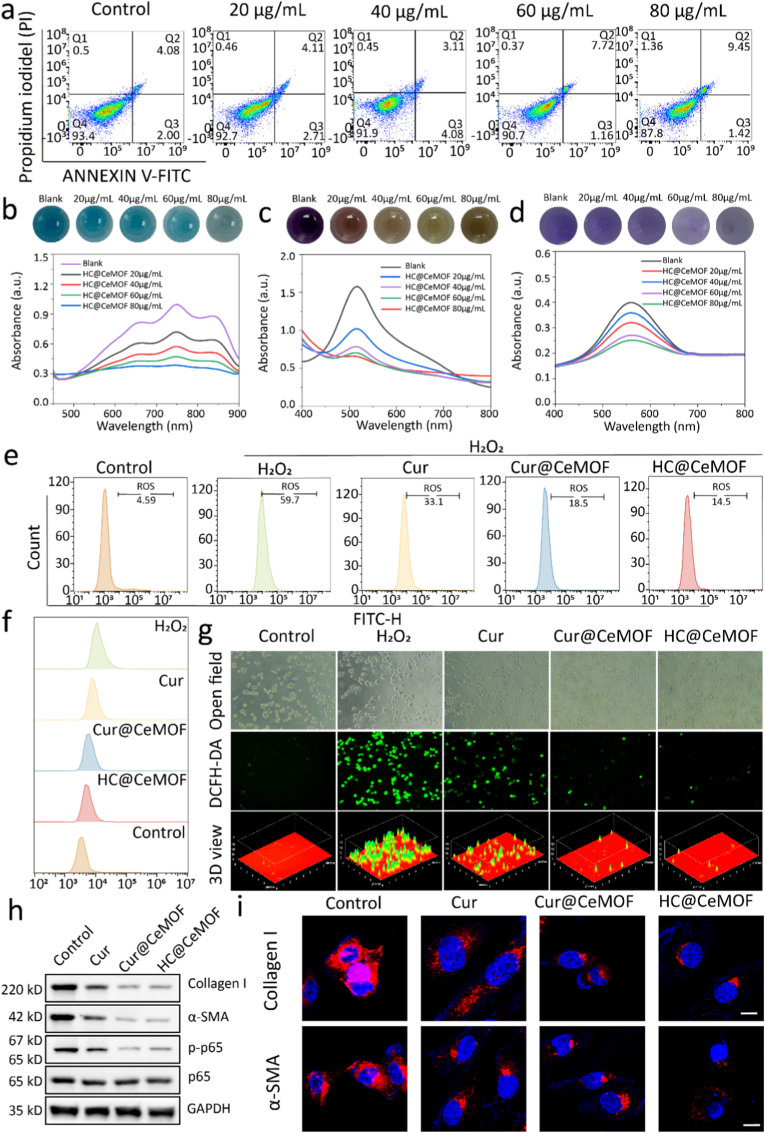
Cellular safety and *in vitro* ROS clearance by nanomaterials. (a) Flow cytometry analyzes the apoptosis rate of HPSCs after co-culturing with HC@CeMOF with different concentrations for one day. (b) ABTS·^+^ scavenging ratio and digital photographs of the scavenging process of HC@CeMOF. (c) DPPH· scavenging ratio and digital photographs of the scavenging process of HC@CeMOF. (d) PTIO· scavenging ratio and digital photographs of the scavenging process of HC@CeMOF. (e) Quantitative analysis of ROS levels in RAW264.7 cells after different treatments. (f) Mountain range plot showing ROS content in RAW264.7 cells after different treatments. (g) The fluorescence images of RAW264.7 cells following various treatments. (h) WB analysis in HPSCs after various treatments. (i) CLSM images are shown for α-SMA and collagen I protein expression in HPSCs after different treatments, bars represent 10 μm.

The safety of nanomaterial is the primary determinant of its potential for clinical translation. To assess the biocompatibility of HC@CeMOF nanozymes for biomedical applications, we first conducted *in vitro* cytotoxicity evaluations. Various concentrations of HC@CeMOF were dispersed in cell culture medium and co-incubated with HPSCs. CCK-8 assays demonstrated that at a concentration of 60 μg/mL, the viability of HPSCs remained above 90 % over a 3-day period (Figure S6). Additionally, flow cytometry was employed to analyze cell apoptosis following co-incubation with the nanozymes. The results revealed a cell survival rate of 90.7 % at 60 μg/mL HC@CeMOF, consistent with the CCK-8 findings (Fig. 3a). Furthermore, live/dead cell staining was performed on HPSCs after 3 days of co-culture with various concentrations of HC@CeMOF. Fluorescence microscopy showed that HC@CeMOF exhibited high cytocompatibility (>85 %) at both 60 μg/mL and 80 μg/mL compared with the control group. 3Dview analysis revealed that the 80 μg/mL group contained more red and purple signals within the green fluorescence regions than the 60 μg/mL group, indicating higher cell death (Figure S7). Based on these results, 60 μg/mL was ultimately selected as the final therapeutic concentration.

Given that the nanozymes in this study were intended for administration via tail vein injection in mice, their hemocompatibility was also evaluated. As shown in Figure S8, with water as the positive control and PBS as the negative control, the hemolysis rates at HC@CeMOF concentrations of 20, 40, 60, and 80 μg/mL were 0.2 %, 0.7 %, 1 %, and 1.2 %, respectively. All values were well below the hemolysis threshold of 5 %, indicating excellent hemocompatibility. Collectively, these results confirm that HC@CeMOF nanozymes possess ideal cytocompatibility and hemocompatibility, supporting their potential for further biomedical research.

Oxidative stress is a key pathological mechanism driving the onset and progression of CP [6]. Therefore, endowing nanomaterials with potent antioxidant capabilities represents a promising therapeutic strategy for this condition. In this study, the total antioxidant capacity of HC@CeMOF was first assessed using the ABTS·^+^ assay. The results showed that the ABTS·^+^ scavenging efficiency increased progressively with rising concentrations of HC@CeMOF (Fig. 3b). By monitoring the characteristic absorption peak of ABTS·^+^ at 734 nm in the presence of HC@CeMOF, a concentration-dependent decrease in absorbance was observed, with a maximum scavenging rate of 52.17 % at 60 μg/mL. Corresponding optical images further confirmed that the solution's color became progressively lighter with increasing HC@CeMOF concentration. Subsequently, the ability of HC@CeMOF to scavenge RNS and ROS was evaluated using the DPPH· (Fig. 3c) and PTIO· assay (Fig. 3d), respectively. Consistent with the ABTS·^+^ findings, higher concentrations of HC@CeMOF led to reduced absorbance peaks of DPPH· at 519 nm and PTIO· at 557 nm. Quantitative analysis revealed that at 60 μg/mL, the scavenging efficiencies for DPPH· and PTIO· reached 55.37 % and 32.1 %, respectively. Optical observations also confirmed a gradual fading of the solution color, indicating the material's effective scavenging activity against both RNS and ROS. To further validate the antioxidant performance of the nanozyme from another perspective, commercial assay kits were used to assess its ability to scavenge hydrogen peroxide (H_2_O_2_) and superoxide anions (O_2_^−^). Results demonstrated that HC@CeMOF could eliminate 69.32 % of H_2_O_2_ and 51.05 % of O_2_^−^ within 30 min (Figure S9 and S10).

Then, under a fixed CeMOF concentration (60 μg/mL), the initial reaction rates were determined by monitoring the change in product absorbance to evaluate its catalytic activity toward hydrogen peroxide. As the H_2_O_2_ concentration increased from 1 to 10 mM, the initial rate rose rapidly and plateaued at higher concentrations. Nonlinear fitting of the data using the Michaelis–Menten equation yielded a correlation coefficient of R^2^ > 0.99, indicating that the catalytic behavior of Ce-MOF follows typical enzyme kinetics. Further, a Lineweaver–Burk plot was constructed by taking the reciprocal of substrate concentration and initial rate, and the data points fell well along a linear regression (Figure S11), further confirming the peroxidase-like catalytic mechanism. From the fitting, the kinetic parameters were determined as K_m_ = 2.57 mM and V_max_ = 197.3 μM s^−1^, indicating high substrate affinity and excellent catalytic efficiency, characteristic of peroxidase-like activity. These findings collectively confirm that HC@CeMOF exhibits outstanding *in vitro* antioxidant activity and holds significant potential in modulating ROS to control the progression of CP.

To further substantiate its antioxidant capability, an *in vitro* inflammatory cell model was established by treating RAW264.7 macrophages with H_2_O_2_ to induce oxidative stress. Here, we chose macrophages as the ROS cell model because they produce high levels of ROS upon stimulation, providing a sensitive system to evaluate ROS-scavenging effects [[44], [45], [46]]. Moreover, macrophages are key inflammatory cells enriched in chronic pancreatitis, where ROS-driven macrophage activation promotes cytokine secretion and subsequent PSC activation [[47], [48], [49]]. Assessing ROS clearance in macrophages clarifies upstream anti-inflammatory mechanisms that ultimately suppress PSC-mediated fibrosis. The cells were then treated with PBS, Cur, Cur@CeMOF, or HC@CeMOF, and intracellular ROS levels were evaluated using the DCFH-DA fluorescent probe via flow cytometry and fluorescence microscopy. Flow cytometric analysis revealed that compared to the positive control, Cur significantly reduced the proportion of ROS-positive cells. Notably, both Cur@CeMOF and HC@CeMOF treatments exhibited stronger antioxidant effects, with HC@CeMOF showing the most pronounced reduction—decreasing ROS-positive cells from 59.7 % to 14.5 % (Fig. 3e and f). In line with these findings, fluorescence microscopy also demonstrated markedly reduced green fluorescence in the HC@CeMOF-treated group, further corroborating its potent intracellular ROS scavenging activity (Fig. 3g). From these results, we can observe that Cur exhibits a certain degree of ROS-scavenging activity compared with the control group, which is consistent with previous reports [50]. Notably, when Cur is loaded into CeMOF, its ROS-scavenging effect is markedly enhanced. This improvement is primarily attributable to the intrinsic reactive oxygen species–eliminating capacity of Ce-based materials, which stems from the Ce^3+^/Ce^4+^ redox cycling and oxygen-vacancy–mediated mechanisms. Their strong antioxidant capability has been extensively validated in earlier studies [32,46,51].

In summary, HC@CeMOF possesses ideal compatibility and exhibits excellent antioxidant properties, effectively eliminates intracellular ROS, and shows significant therapeutic potential for the treatment of CP.

### 3.4 *In vitro* antifibrotic effects of HC@CeMOF

Fibrosis represents the most prominent pathological feature of CP, leading to progressive impairment of both exocrine and endocrine pancreatic functions [52]. This is also a major factor contributing to the clinical difficulty in curing CP. PSCs serve as key effector cells in response to various pancreatic injuries. Under persistent inflammatory stimulation, PSCs become activated and secrete abundant ECM proteins, including collagen, α-SMA, fibronectin, and tissue repair proteins [2]. These factors drive the pancreas toward chronic and pathological fibrosis. Therefore, timely alleviation of inflammation and inhibition of PSC activation are crucial strategies for controlling or even reversing pancreatic fibrosis. Given that we have previously confirmed the excellent antioxidant properties of HC@CeMOF *in vitro*, we hypothesized that HC@CeMOF may exert anti-inflammatory and anti-fibrotic effects by mitigating oxidative stress. To investigate the antifibrotic capacity of HC@CeMOF *in vitro*, we co-cultured HPSCs with Cur, Cur@CeMOF, and HC@CeMOF for 24 h. WB analysis showed that, compared with the control group, Cur treatment led to reduced expression levels of αSMA and Collagen I, while treatment with Cur@CeMOF and HC@CeMOF resulted in a more pronounced downregulation of both markers (Fig. 3h and Figure S12). To further validate these findings, we performed immunofluorescence staining to examine the expression of αSMA and Collagen I following different treatments. The results revealed that HC@CeMOF significantly suppressed the expression of both proteins compared to the control and Cur groups and showed slightly lower expression than the Cur@CeMOF group, consistent with the WB results (Fig. 3i).

Additionally, we evaluated the effect of different treatments on the NF-κB signaling pathway, a key regulator of inflammation. The results demonstrated that Cur, Cur@CeMOF, and HC@CeMOF all reduced the expression of phosphorylated P65 relative to total P65 (p-P65/p65), with HC@CeMOF exhibiting the most substantial downregulation (Fig. 3h). This enhancement can be attributed to two factors: (i) the intrinsic ROS-scavenging activity of CeMOF, which reduces oxidative stress and upstream NF-κB activation; and (ii) the sustained release of Cur from CeMOF, which prolongs intracellular drug exposure and allows Cur to exert a stronger inhibitory effect on NF-κB signaling. The improved therapeutic efficacy through sustained drug release is a known advantage of MOF-based nanocarriers, consistent with previous studies. This indicates that HC@CeMOF has the potential to effectively modulate inflammation by suppressing the activation of the NF-κB pathway.

Collectively, these findings indicate that HC@CeMOF possesses potent antioxidant activity while inhibiting the NF-κB inflammatory signaling pathway, and the synergy of these effects can effectively prevent PSCs from transitioning to a fibrotic phenotype.

### 3.5 Biocompatibility analysis of HC@CeMOF

**Fig. 4:**
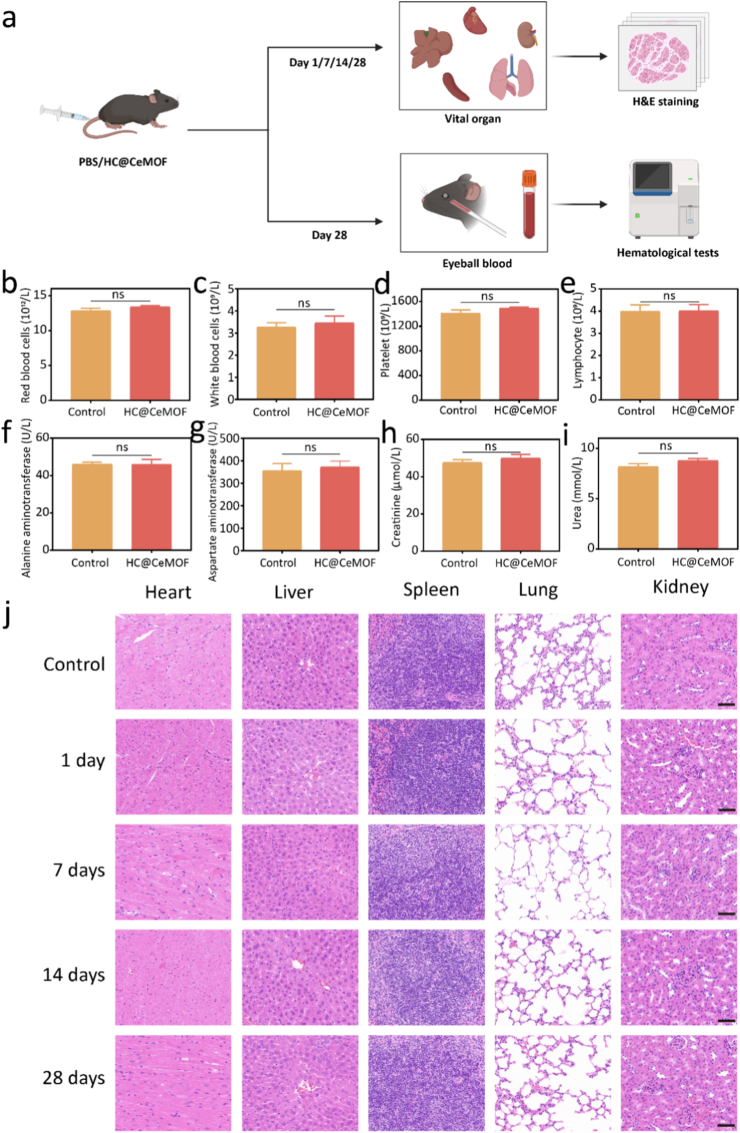
Animal safety assessment after nanomaterials treatment. (a) Schematic diagram of animal safety assessment. (b–e) Routine blood tests in mice after HC@CeMOF treatment. (f–i) Liver and kidney function assays in mice after HC@CeMOF treatment. (j) H&E staining of the heart, liver, spleen, lung, and kidney of mice after treatment with HC@CeMOF within 28 days, bars represent 50 μm. Data are presented as the mean ± SD (n = 3), ns: not significant.

Before further applying HC@CeMOF, it is essential to comprehensively assess its potential nanotoxicity *in vivo*. As shown in Fig. 4a, healthy mice were randomly assigned to a control group and an HC@CeMOF treatment group. Mice received daily intravenous injections of either PBS or HC@CeMOF via the tail vein. On days 1, 7, 14, and 28, mice were euthanized, and major organs—including the liver, heart, lungs, spleen, and kidneys—were collected for H&E staining. In addition, blood samples were collected from the orbital sinus on day 28 for complete blood count as well as hepatic and renal function tests.

The results showed no significant differences in red blood cell (Fig. 4b), white blood cell (Fig. 4c), platelet (Fig. 4d), or lymphocyte counts (Fig. 4e) between the HC@CeMOF-treated group and the control group after 28 days of treatment. Similarly, serum biochemical analyses indicated that liver function markers (AST, ALT) and renal function markers (BUN, CRE) in the HC@CeMOF group were comparable to those in the control group (Fig. 4f–i). Histopathological examinations of major organs in the HC@CeMOF-treated mice revealed no pathological abnormalities in the heart, liver, spleen, lungs, or kidneys at any of the examined time points (days 1, 7, 14, or 28), indicating that the nanozymes did not induce tissue damage in these organs (Fig. 4j). Collectively, these findings demonstrate that HC@CeMOF exhibits excellent biocompatibility and biosafety, making it well-suited for subsequent *in vivo* therapeutic applications in mice.

### 3.6 Targeting ability of HC@CeMOF

Due to the deep anatomical location of the pancreas and its encapsulation by several organs such as the stomach, duodenum, and spleen, along with its relatively isolated and poorly vascularized network, and the presence of fibrosis and vascular abnormalities under pathological conditions, both orally and intravenously administered drugs face significant challenges in reaching the lesion site and maintaining effective therapeutic concentrations [4,53]. Therefore, enabling nanomaterials to specifically target pancreatic lesions holds substantial significance for improving the therapeutic efficacy in CP. It is well established that macrophages play a central role in the development and progression of CP, and are therefore abundantly present at the site of inflammation.

**Fig. 5:**
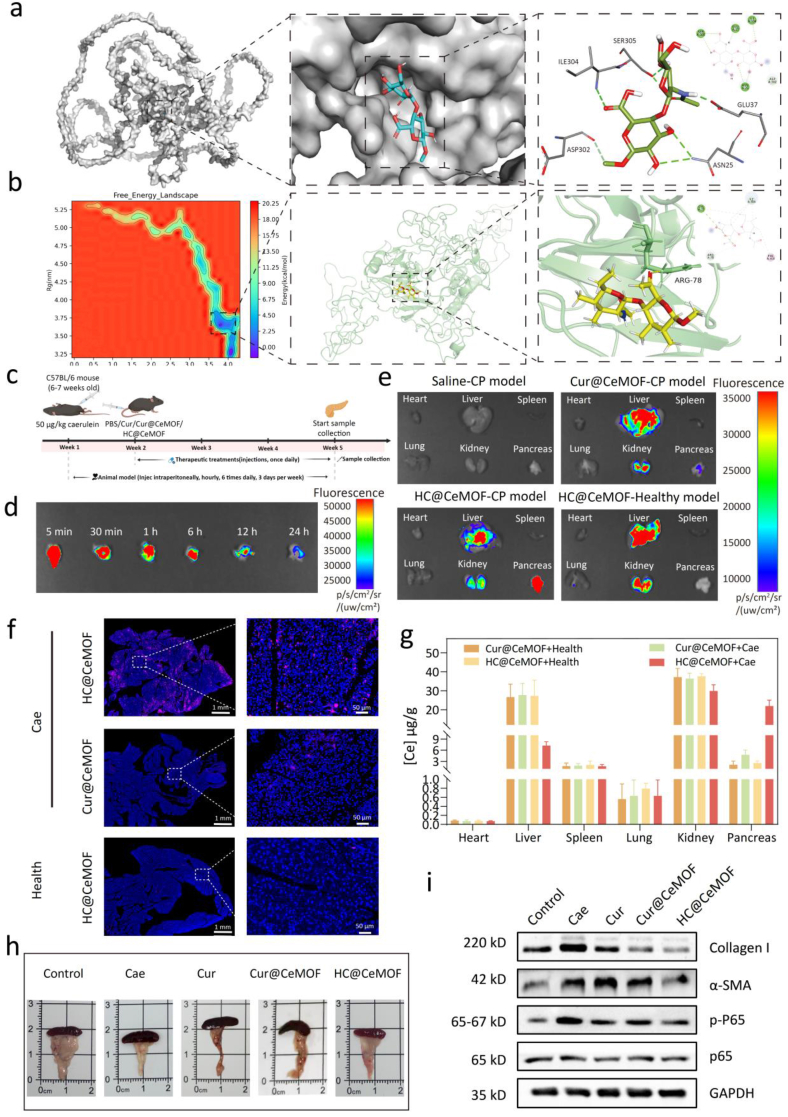
Evaluation of nanomaterials pancreatic accumulation capacity and *in vivo* therapeutic efficacy. (a) Diagram of the interaction between HA and CD44 protein. (b) The free energy landscape and interaction conformations of protein CD44 and small molecule hyaluronic acid in molecular dynamics. (c) Schematic diagram of the construction and treatment process for the CP mouse model. (d) Temporal accumulation changes of Cy5.5-labeled HC@CeMOF in pancreatic tissues of CP mice following tail vein injection using the IVIS imaging system. (e) The bio-distribution of Cy5.5-labeled HC@CeMOF and Cy5.5-labeled Cur@CeMOF in CP mice and health mice using the IVIS imaging system. (f) Fluorescent images of the pancreas of healthy mice and CP mice after incubation Cy5.5-labeled nanomaterials. Red represents nanomaterials and blue represents nuclei. Bars represent 1 mm; Enlarged images: bars represent 50 μm. (g) The distribution of Ce in various tissues of mice was analyzed by ICP. (h) Optical images of the pancreas in mice after different treatments. (i) WB analysis conducted on the expression levels of pancreatic fibrotic proteins in mice after different treatments (n = 3). (For interpretation of the references to color in this figure legend, the reader is referred to the Web version of this article.)

To achieve targeted delivery to pancreatic lesions, this study engineered the surface of nanomaterials with HA, leveraging the specific binding affinity between HA and CD44, a receptor that is overexpressed on macrophages within the inflammatory microenvironment. We first performed molecular docking to assess the binding affinity between HA and CD44 on the macrophage surface. The results demonstrated strong binding potential, with a binding energy of −5.47 kcal/mol. Analysis of the binding interface revealed that key residues including ILE304, SER305, ASP302, GLU37, and ASN25 are primarily involved in HA–CD44 interactions, with hydrogen bonding serving as the dominant interaction mode (Fig. 5a). These findings suggest that HA can stably bind to CD44.

Furthermore, molecular dynamics (MD) simulations were conducted to investigate the dynamic binding behavior of HA and CD44 under physiological conditions. The RMSD analysis indicated that a stable binding conformation was achieved at 62 ns (Figure S13a and S13b). The radius of gyration (Rg) analysis demonstrated increasingly tighter interactions between HA and CD44 over time (Figure S13c). RMSF results showed pronounced fluctuations at residues Val79, Phe378, and Glu379, with peak RMSF values reaching 4.017, 5.348, and 6.113, respectively (Figure S13d). After removing periodicity from the MD trajectory, MM/PBSA calculations were performed on the complex during the 80–100 ns window, revealing a total binding free energy of −26.81 kcal/mol (Figure S14a). Under physiological conditions, HA was found to primarily interact with residues CYS77, ILE304, ARG78, and PHE303 (Figure S14b). Structural analysis of representative MD conformations further confirmed that HA forms hydrogen bonds with CYS77, ILE304, and ARG78, while also engaging in Pi-Alkyl and Alkyl interactions with PHE303 (Fig. 5b). Collectively, these results indicate that HA forms a stable binding complex with CD44 on the macrophage surface, suggesting that HA-modified nanomaterials possess macrophage-targeting capabilities.

Based on these findings, we next evaluated the *in vivo* targeting ability of HC@CeMOF in a CP mouse model. CP was induced via repeated caerulein administration (Fig. 5c). Cy5.5-labeled HC@CeMOF was administered via tail vein injection, and pancreatic tissues were collected at various time points for analysis. IVIS imaging revealed that the labeled nanomaterials accumulated extensively in the pancreas as early as 5 min post-injection and persisted for at least 24 h (Fig. 5d and Figure S15). We further assessed the biodistribution of the nanomaterials 2 h after injection. In the CP model, IVIS imaging showed that non-HA-modified Cur@CeMOF primarily accumulated in the liver and kidneys, with minimal pancreatic localization. In contrast, HA-modified HC@CeMOF demonstrated pronounced accumulation in the pancreas with reduced hepatic and renal distribution. In healthy mice, HC@CeMOF showed negligible pancreatic accumulation (Fig. 5e and Figure S16). Frozen sections of pancreatic tissue further confirmed these results under fluorescence microscopy. Abundant punctate red fluorescence signals were observed in the pancreas of CP mice treated with HC@CeMOF, whereas only sparse signals were present in the Cur@CeMOF group (Fig. 5f and Figure S17). Additionally, inductively coupled plasma atomic emission spectroscopy (ICP-AES) was used to quantify the distribution of Ce across various organs. As shown in Fig. 5g, Ce primarily accumulated in the liver and kidneys of healthy mice. However, in CP mice, significant Ce enrichment was observed only in the pancreas of the HC@CeMOF group. To further validate the role of HA in this process, we pre-injected free HA into CP mice 30 min before administering HC@CeMOF. IVIS imaging showed that, compared with the saline-preinjected group, mice receiving free HA exhibited a markedly reduced pancreatic fluorescence signal. Consistently, ICP analysis of pancreatic Ce content also demonstrated substantially lower Ce levels in the free HA group than in the saline group. These results indicate that HA is the key factor conferring the targeting capability of HC@CeMOF (Figure S18).

Here, we selected HA as the targeting ligand for several reasons. First, compared with other antibody-based or synthetic polymeric materials, HA offers superior biocompatibility, non-immunogenicity, and suitability for long-term administration [54]. Second, its CD44-mediated affinity toward inflammatory macrophages in chronic pancreatitis confers strong targeting capability, which is consistent with both our experimental findings and prior studies [55,56]. Moreover, HA-coated nanomaterials exhibit favorable system stability and are easier to manufacture on a large scale, supporting future clinical translation [54]. Finally, HA coating can partially improve the hydrophilicity and colloidal stability of the nanoparticles, reducing plasma protein adsorption and opsonin binding, thereby decreasing the likelihood of rapid recognition and clearance by the mononuclear phagocyte system in the liver and spleen. It is worth noting that after HC@CeMOF enters the bloodstream, a portion of the nanoparticles may undergo nonspecific interactions with serum proteins. However, accurately determining the extent of this binding would require large-scale proteomic analyses and detailed pharmacokinetic studies. Moreover, our results have already demonstrated that the majority of HC@CeMOF is efficiently accumulated at the lesion site. Therefore, we did not further investigate this aspect. Based on the findings above, we can still conclude that HC@CeMOF exhibits a strong and specific targeting capability toward pancreatitis-associated pancreatic lesions, providing robust experimental support for its potential as an effective therapeutic agent for chronic pancreatitis.

### 3.7 *In vivo* therapeutic effect of CP

Subsequently, we evaluated the therapeutic efficacy of the nanomaterial *in vivo* against CP. As shown in Fig. 5c, beginning from the second week after CP model induction until the day before sacrifice (a total of 21 days), mice were administered various treatments via daily tail vein injection. Compared with the control group, mice in the Cae group exhibited a pronounced decline in body weight over time. In contrast, mice treated with Cur, Cur@CeMOF, or HC@CeMOF showed partial improvement in body weight, with the most significant recovery observed in the HC@CeMOF group (Figure S19), indicating the most evident disease remission in this group. At the end of the treatment period, the mice were sacrificed, and gross examination of the pancreas revealed that CP-induced mice showed substantial pancreatic atrophy and hardening compared to controls. Drug treatment partially restored pancreatic morphology, with the HC@CeMOF group exhibiting the most pronounced improvement (Fig. 5h). Quantification of pancreatic weight showed that the HC@CeMOF group had the heaviest pancreata among CP groups, although still lighter than the healthy control, and significantly heavier than those in the Cae group. Meanwhile, the pancreatic weights in the Cur and Cur@CeMOF groups did not differ significantly from the Cae group (Figure S20). These findings indicate that repeated Cae administration leads to pancreatic atrophy, body weight loss, and reduced pancreatic mass in mice, all of which can be effectively alleviated by HC@CeMOF treatment. Additionally, we measured serum amylase and lipase levels in mice after treatment, and the results showed that HC@CeMOF treatment markedly reduced serum amylase levels while increasing serum lipase levels in mice, compared with the other treatment groups (Figure S21). This phenomenon may be attributed to the distinct secretion cycles of amylase and lipase, as well as the acinar atrophy observed in chronic pancreatitis mice, which leads to insufficient lipase secretion. These findings are consistent with previous report [17]. Compared with the control group, serum amylase remained slightly higher and serum lipase slightly lower after HC@CeMOF treatment, although neither difference reached statistical significance. These trends provide supportive evidence for the potential therapeutic benefit of HC@CeMOF in CP.

**Fig. 6:**
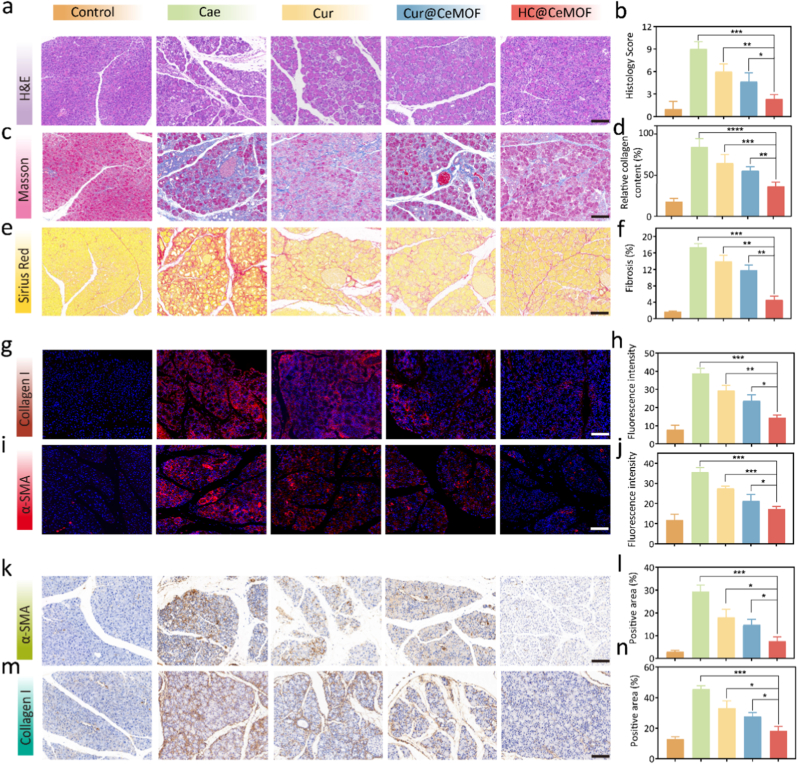
Pathological changes of the pancreas in mice after different treatments. (a) Representative pancreatic H&E staining after different treatment. (b) Histology score of H&E staining. (c) Representative pancreatic Masson staining after different treatment. (c) Quantitative analysis of the Masson staining. (d) Representative pancreatic Sirius Red staining after different treatment. (f) Quantitative analysis of the Sirius Red staining. (g–j) Representative pancreatic immunofluorescence staining and corresponding quantitative analysis to evaluate pancreatic fibrosis biomarkers (α-SMA and Collagen I) after different treatment. (k–n) Representative pancreatic immumohistochemical staining and corresponding quantitative analysis to evaluate pancreatic fibrosis biomarkers (α-SMA and Collagen I) after different treatment. Bars represent 100 μm. Data are presented as the mean ± SD (n = 3), ∗*p* < 0.05, ∗∗*p* < 0.01, ∗∗∗*p* < 0.001, ∗∗∗∗*p* < 0.0001. (For interpretation of the references to color in this figure legend, the reader is referred to the Web version of this article.)

To assess the anti-fibrotic and anti-inflammatory effects of HC@CeMOF, we examined the expression levels of fibrotic markers α-SMA and Collagen I in the pancreas via WB analysis. The results revealed that all drug treatments reduced the expression of these fibrotic proteins in CP mice, with HC@CeMOF showing the most significant downregulation (Fig. 5i and Figure S22). Notably, the *in vivo* anti-fibrotic effect of Cur@CeMOF was less pronounced than *in vitro*, and inferior to that of HC@CeMOF. This discrepancy may be attributed to the pancreatic-targeting capability of HC@CeMOF, which facilitates its accumulation at the lesion site and significantly enhances the bioavailability of the therapeutic agent. To further confirm the *in vivo* anti-fibrotic efficacy of HC@CeMOF, we performed histopathological assessments of pancreatic tissue, including H&E, Masson, and Sirius Red staining (Fig. 6a–f). The control group displayed normal pancreatic architecture without signs of fibrosis. In contrast, CP mice exhibited widespread acinar atrophy, inflammatory infiltration, and diffuse stromal fibrosis. Treatment with Cur or Cur@CeMOF slightly alleviated acinar atrophy and fibrosis, whereas HC@CeMOF treatment markedly reversed CP-associated pathological features, including acinar cell atrophy, ECM accumulation, ductal dilation, and immune cell infiltration.

Furthermore, we evaluated the expression of α-SMA and Collagen I in pancreatic tissues using immunofluorescence and immunohistochemistry. As shown in Fig. 6g–n, α-SMA and Collagen I were highly expressed in the pancreata of Cae-induced CP mice, whereas their expression levels were significantly reduced following HC@CeMOF treatment, reaching levels comparable to those in the control group.

Taken together, these data suggest that HC@CeMOF effectively alleviates Cae-induced CP severity *in vivo* by scavenging ROS and suppressing fibrotic marker expression. Its therapeutic performance significantly surpasses that of Cur and Cur@CeMOF, which can be attributed to its precise targeting of CP lesions.

### 3.8 The ability of HC@CeMOF to regulate inflammatory factors *in vivo*

**Fig. 7:**
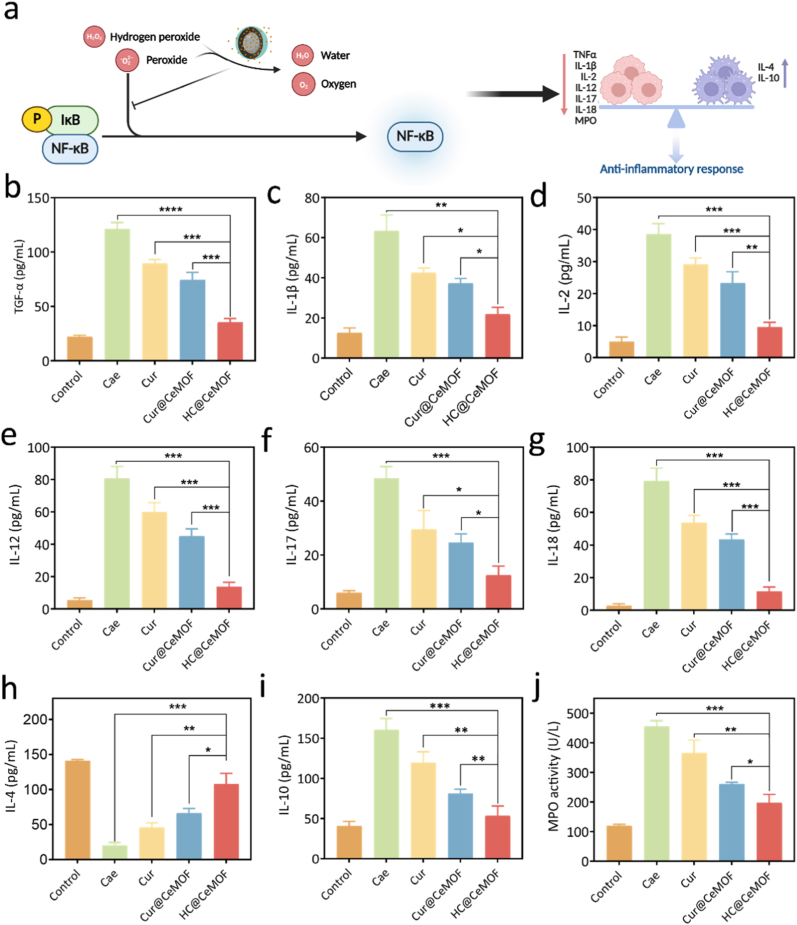
*In vivo* anti-inflammatory evaluation of HC@CeMOF. (a) Diagram of the anti-inflammatory mechanism of HC@CeMOF *in vivo*. (b–j) The expression levels of inflammatory factors in mice after different treatments were measured by ELISA. Data are presented as the mean ± SD (n = 3), ∗*p* < 0.05, ∗∗*p* < 0.01, and ∗∗∗*p* < 0.001.

Next, we further investigated the *in vivo* therapeutic mechanism of HC@CeMOF in CP. CP is characterized by extensive proliferation of PSCs and deposition of ECM, resulting in the loss of acinar cells and progressive pancreatic fibrosis [5]. Accumulation of ROS has been recognized as a central trigger for PSC activation, which plays a pivotal role in the development of pancreatic fibrosis [57]. Our previous *in vitro* studies confirmed that HC@CeMOF can effectively scavenge ROS while inhibiting NF-κB signaling, thereby suppressing the activation of PSCs. WB analysis of pancreatic tissue protein expression further validated these findings: caerulein administration upregulated NF-κB/p65 expression, whereas HC@CeMOF treatment effectively reversed this elevation in CP mice (Fig. 5i and Figure S22). Based on these results, we hypothesize that the mechanism by which HC@CeMOF alleviates CP involves efficient ROS scavenging, suppression of NF-κB/p65 expression, subsequent modulation of inflammatory cytokine profiles, and ultimately attenuation of pancreatic fibrosis (Fig. 7a).

To assess the cytokine expression profiles in pancreatic tissue, we measured the levels of pro-inflammatory and anti-inflammatory cytokines in CP mice using ELISA after treatment with the nanomaterials. The results demonstrated that caerulein injection significantly increased the expression of pro-inflammatory cytokines (TNF-α, IL-1β, IL-2, IL-12, IL-17, and IL-18) (Fig. 7b–g) while decreasing the levels of anti-inflammatory cytokines (IL-4 and IL-10) (Fig. 7h–i), confirming a pronounced inflammatory response during CP induction. Treatment with Cur, Cur@CeMOF, and HC@CeMOF suppressed pro-inflammatory cytokines and upregulated anti-inflammatory cytokines, indicating that all three formulations could modulate the inflammatory response. Among them, HC@CeMOF showed the most pronounced regulatory effect on cytokine expression, which may be attributed to its hyaluronic acid-mediated targeting ability that enhances accumulation at CP lesions, thereby improving the *in vivo* bioavailability of the nanomaterial. Additionally, we evaluated the expression of myeloperoxidase (MPO), a key enzyme secreted predominantly by activated neutrophils, whose activity reflects neutrophil proliferation and tissue damage severity and serves as an indicator of inflammation in CP. The results showed that all three treatments (Cur, Cur@CeMOF, and HC@CeMOF) significantly reduced MPO levels compared to the Cae group, with HC@CeMOF exhibiting the greatest suppression, outperforming both Cur and Cur@CeMOF groups (Fig. 7j).

In conclusion, HC@CeMOF scavenges excessive ROS in the tissue through its enzyme-mimetic activity, thereby alleviating oxidative stress. This redox modulation is associated with reduced inflammatory cytokine and chemokine levels, which may contribute to a less pro-fibrotic microenvironment. Concurrently, HC@CeMOF downregulates NF-κB expression. Together, these effects are correlated with suppressed PSC activation and reduced collagen deposition. However, the causal relationships among macrophage modulation, PSC regulation, and fibrosis attenuation warrant further investigation.

## 4 Conclusion

CP is a lifelong progressive fibrotic inflammatory disease with an increasing incidence worldwide, posing a substantial medical and economic burden. In this study, we innovatively developed a potent antioxidant and targeted nanoplatform (HC@CeMOF) by integrating the natural polyphenolic antioxidant Cur with small-sized CeMOF nanomaterials, further coated with HA. Our findings confirmed that HC@CeMOF possesses excellent cellular safety and biocompatibility. In a CP mouse model, leveraging the inflammation-targeting capability of HA, HC@CeMOF preferentially accumulated at the sites of CP inflammation. Subsequently, through the synergistic effects of Cur and CeMOF, it effectively scavenged ROS at the lesion site and downregulated NF-κB expression, thereby modulating the local inflammatory cytokine microenvironment and ultimately achieving a therapeutic effect against pancreatic fibrosis. In summary, the HC@CeMOF nanoplatform developed in this study integrates targeted drug delivery with potent ROS scavenging and demonstrates promising therapeutic efficacy for CP, offering a potential strategy for future intervention and treatment of this challenging disease.

## CRediT authorship contribution statement

**Yongkang Lai:** Writing – original draft, Methodology, Investigation, Funding acquisition, Data curation, Conceptualization. **Yongliang Ouyang:** Writing – original draft, Methodology, Investigation, Formal analysis, Data curation. **Xiaojing Yin:** Writing – original draft, Validation, Methodology, Investigation, Formal analysis, Data curation. **Tao Yu:** Visualization, Resources, Funding acquisition. **Jianhua Wan:** Validation, Investigation, Formal analysis. **Xueyang Li:** Visualization, Validation, Investigation. **Yi Hu:** Writing – review & editing, Supervision, Project administration, Funding acquisition. **Xu Shu:** Writing – review & editing, Supervision, Resources, Project administration, Conceptualization. **Huan Wang:** Writing – review & editing, Supervision, Project administration, Investigation, Funding acquisition.

## Ethics approval and consent to participate

All animal procedures in this study were approved by the Ethics Committee of the First Affiliated Hospital of Nanchang University (Nanchang, Jiangxi Province, China, Approval Number: CDYFY-IACUC-202505GR063).

## Funding

This work was supported by the 10.13039/501100001809National Natural Science Foundation of China (NO. 82400657, 82000531, 82360118 and 82570666), 10.13039/501100004479Natural Science Foundation of Jiangxi Province (20242BAB25436), Jiangxi Provincial 10.13039/100018696Health Technology Project (202410174 and 202310154), Jiangxi Province Traditional Chinese Medicine Science and Technology Project (2024B0036 and 2024B0280), Young Scholar Project of the First Affiliated Hospital of 10.13039/501100004637Nanchang University (YFYPY202534), Science and Technology Project of the 10.13039/501100020205Health Commission of Jiangxi Province (202610026).

## Declaration of competing interest

The authors declare that they have no known competing financial interests or personal relationships that could have appeared to influence the work reported in this paper.

## Data Availability

Data will be made available on request.
